# Estimation of crop plant density at early mixed growth stages using UAV imagery

**DOI:** 10.1186/s13007-019-0449-1

**Published:** 2019-06-19

**Authors:** Joshua C. O. Koh, Matthew Hayden, Hans Daetwyler, Surya Kant

**Affiliations:** 1 Agriculture Victoria, Grains Innovation Park, 110 Natimuk Rd, Horsham, VIC 3400 Australia; 2 Agriculture Victoria, AgriBio, Centre for AgriBioscience, 5 Ring Road, Bundoora, VIC 3083 Australia; 3 School of Applied Systems Biology, La Trobe University, Bundoora, VIC 3086 Australia

**Keywords:** Object-based image analysis, Plant phenotyping, Safflower, Seedling count, Unmanned aerial vehicle

## Abstract

**Background:**

Unmanned aerial vehicles (UAVs) equipped with lightweight sensors are making a significant impact in field-based crop phenotyping. UAV platforms have been successfully deployed to acquire phenotypic data in a precise and efficient manner that would otherwise be time-consuming and costly to acquire when undertaken through manual assessment. One example is the estimation of plant density (or counts) in field experiments. Challenges posed to digital plant counting models are heterogenous germination and mixed growth stages that are present in field experiments with diverse genotypes. Here we describe, using safflower as an example, a method based on template matching for seedling count estimation at early mixed growth stages using UAV imagery.

**Results:**

An object-based image analysis algorithm based on template matching was developed for safflower seedling detection at early mixed growth stages in field experiments conducted in 2017 and 2018. Seedling detection was successful when tested using a grouped template type with 10 subgroups representing safflower at 2–4 leaves growth stage in 100 selected plots from the 2017 field experiment. The algorithm was validated for 300 plots each from the 2017 and 2018 field experiments, where estimated seedling counts correlated closely with manual counting; R^2^ = 0.87, MAE = 8.18, RSME = 9.38 for 2017 field experiment and R^2^ = 0.86, MAE = 9.16, RSME = 10.51 for 2018.

**Conclusion:**

A method for safflower seedling count at early mixed growth stages using UAV imagery was developed and validated. The model performed well across heterogenous growth stages and has the potential to be used for plant density estimation across various crop species.

## Background

Technological advances in the development of unmanned aerial vehicles (UAVs) equipped with sensors are rapidly transforming the discipline of field-based crop phenotyping [1, 2]. UAVs can acquire images with high spatial and temporal resolution for crop variation detection and quantification. In addition, they are flexible in the acquisition time without being limited by ground conditions which may otherwise impede access by human operators and ground-based systems. UAVs equipped with a range of sensors such as optical digital RGB (red, green, blue), multispectral, hyperspectral, thermal and light detection and ranging (LiDAR) have been deployed successfully to estimate biomass, height, nitrogen usage and canopy temperatures in crop plants [3–6]. More recently, UAV-acquired high resolution RGB imagery was used to estimate wheat plant density [7] and rapeseed stand count [8]. Thus, UAV platforms offer novel opportunities to estimate plant density in a high-throughput manner. However, the performance of plant counting models is significantly impacted by crop growth stages, with different estimates observed at early growth stages [7, 8]. For example, seedling count estimation in rapeseed at the two-leaf growth stage based on a multi-regression model differed significantly for two sampling time points [8]. This becomes an issue when heterogenous germination results in mixed growth stages in a field experiment; something often seen in crops with a relatively minor or short breeding history due to varied germination rates between genotypes, and in experiments with diverse genotypes or different treatments, such as varying watering or nutrient regimes.

Using safflower (*Carthamus tinctorius* L.) as an example, this study aimed to develop a method to estimate plant count/density under mixed growth stages post emergence using UAV-acquired high resolution RGB imagery. Safflower is a minor oilseed crop with global seed production of 948,516 tonnes in 2016, equating to 0.28% of world soybean production [9]. However, the development of a genetically engineered safflower cultivar with seed oil that contains approximately 92% oleic acid—named Super High Oleic (SHO) safflower—by the Commonwealth Scientific and Industrial Research Organisation in collaboration with the Grains Research and Development Corporation [10] and subsequent licensing of the SHO technologies and materials to an Australian clean technology company, GO Resources, has paved the way for the establishment of a new oilseeds industry [11]. Breeding efforts for elite SHO safflower cultivars will be accelerated via genome-assisted breeding propelled by high-throughput field phenotyping [12].

We present in detail the image processing and analysis pipeline for the estimation of safflower seedling count at mixed early growth stages based on an object-based image analysis (OBIA) algorithm.

## Methods

### Field experiments

**Fig. 1:**
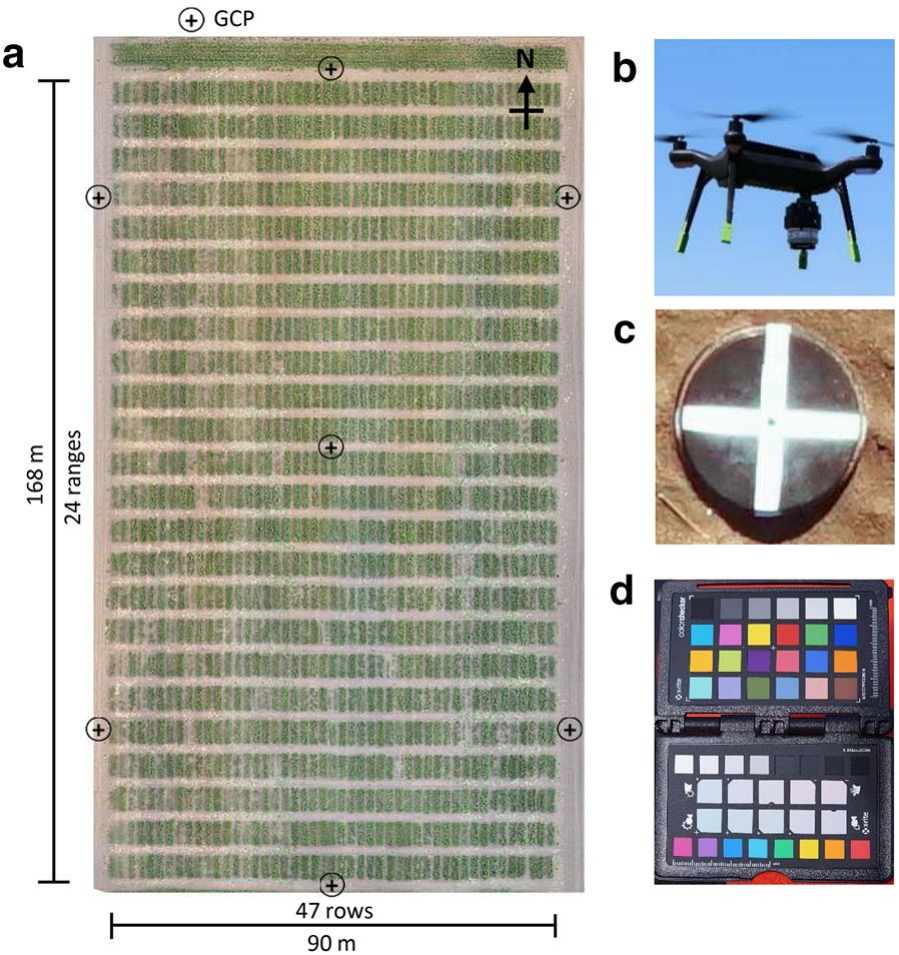
Safflower field experiment. **a** 2017 field experiment design and layout with ground control point (GCP) distribution; the 2018 field experiment had a similar layout, **b** 3DR Solo UAV with a Sony QX1 digital RGB camera attached, **c** GCP, and **d** colour target

Two field experiments were conducted at the Plant Breeding Centre, Agriculture Victoria, Horsham, Victoria, Australia during the winter–spring cropping seasons of 2017 (Lat: 36°44′14.88″S Long: 142°6′51.73″E) (Fig. 1a) and 2018 (Lat: 36°44′14.98″S Long: 142°6′48.80″E). Safflower genotypes were planted in individual plots measuring 5 m long and 1 m wide (5 m^2^). Seeds were sown to achieve a planting density of 40 plants/m^2^ according to the recommended density of 20–40 plants/m^2^ for safflower in Australia [13]. The development and optimization of the OBIA algorithm for safflower seedling detection was conducted on a subset of 100 plots from the 2017 experiment, while for the validation of the algorithm, additional 300 plots each from the 2017 and 2018 field experiments were used. These plots were representative of the overall genotypic diversity and germination heterogeneity observed in the field experiments. Plant count for plots were obtained by manual counting in the field by experts (agriculture scientists), these were further verified digitally by visual counting of seedlings in aerial plot images (R^2^ = 0.94).

### UAV image acquisition

A 3DR Solo (3D Robotics Inc., USA) UAV was used with a custom fixed gimbal to attach a Sony ILCE-QX1 RGB camera with an APS-C type sensor (23.2 × 15.4 mm) and 20.1 megapixels resolution (Fig. 1b). Flight planning and automatic mission control was performed using the android application ‘Tower’. The camera was equipped with a 30 mm focal length lens and set to continuous shooting mode in JPEG format with shutter priority using the Sony PlayMemories android application, resulting in approximately two images captured per second (~ 0.5 Hz frequency). Images were acquired at a flight altitude of 20 m at constant speed of 3.0 ms^−1^ and with an overlap of more than 75% under overcast conditions.

Flights were conducted over the safflower emergence period and data presented in this study corresponded to the period when the majority of safflower plants were at the 2–4 leaves growth stage. Seven black plastic panels of 38 cm diameter and painted with white cross were distributed in the field experiment to serve as ground control points (GCPs) for accurate geo-positioning of images (Fig. 1c). A real-time kinematic global positioning system (RTK-GPS) receiver EMLID Reach RS (https://emlid.com) was used to record the centre of each panel with < 1 centimetre accuracy. An image of a colour target (X-rite ColourChecker Passport, www.xrite.com, Fig. 1d) was captured before and after each flight for white balance correction.

### Image pre-processing and orthomosaic

White balance correction for acquired images was performed in Adobe Lightroom CC and the images were geo-tagged using the 3DR Solo flight log in the freeware, GeoSetter version 3.4. Images were then imported into Pix4D Mapper version 4.2 to generate orthomosaic image, with the coordinates of the GCPs used for georectification. Orthomosaic RGB images generated for the safflower experiments had a ground sampling distance (GSD) of approximately 0.19 cm/pixel.

### OBIA algorithm for safflower detection

**Fig. 2:**
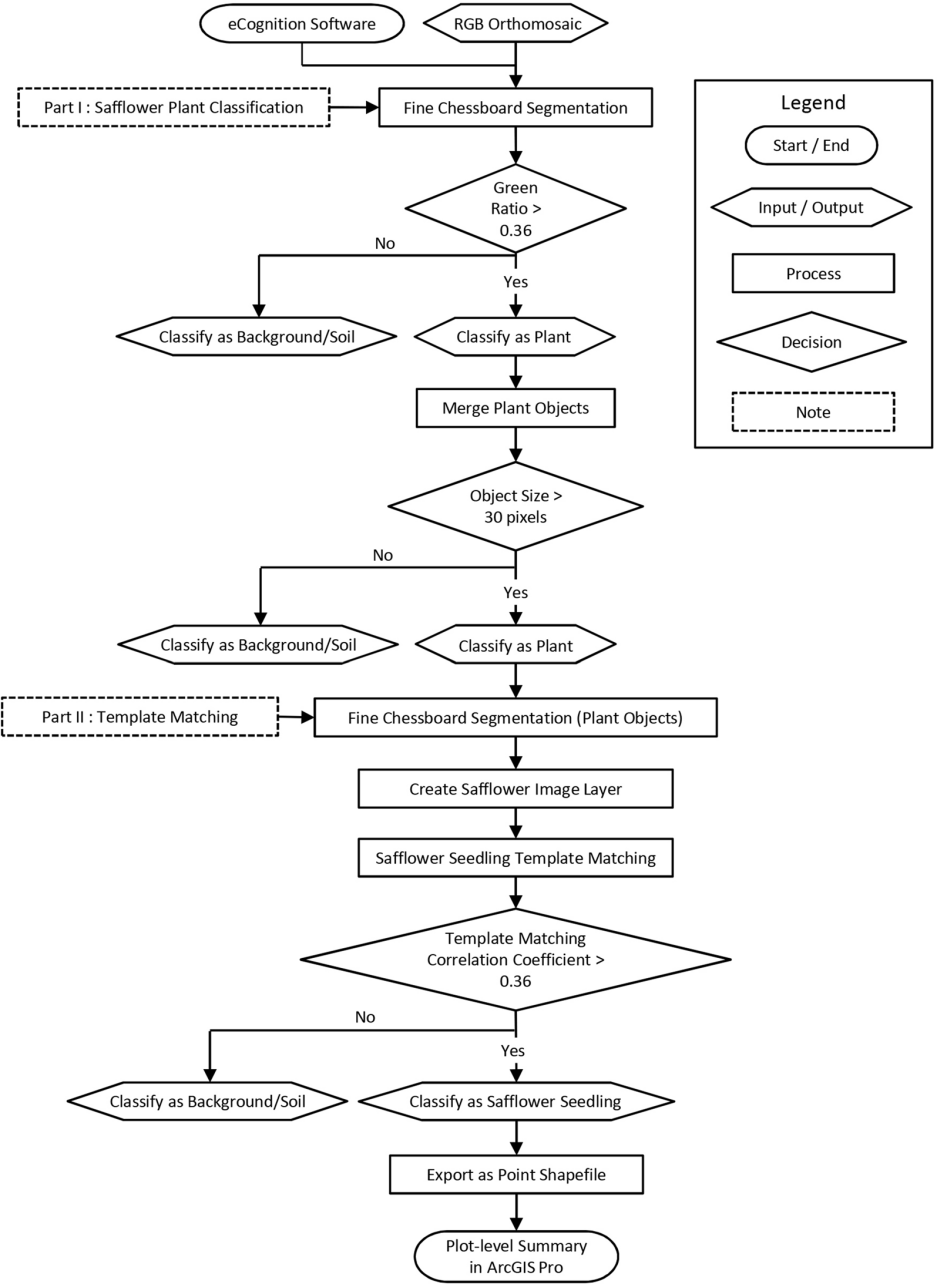
Flowchart of the OBIA algorithm for safflower plant classification and seedling detection. The OBIA algorithm consists of two main parts: safflower plant classification and seedling template matching. The final product is a point shapefile corresponding to safflower seedlings which is imported into the ArcGIS Pro software

The orthomosaic image was imported into eCognition Developer software version 9.3 (http://www.ecognition.com/) for further processing. A fully automated OBIA algorithm for safflower plant classification and seedling detection at early growth stages was developed using eCognition (Fig. 2). The procedure presents an innovative application of the template matching algorithm where areas of an image are matched to a template image, in this case a safflower seedling. To enhance safflower seedling detection accuracy, a grouped template consisting of multiple template subgroups was applied. The grouped template was generated in eCognition’s template editor using 1000 image patches (20 × 20 pixels each) representing safflower seedlings at early growth stages of 2–4 leaves selected from 100 plots in the 2017 field experiment. In addition, the grouped template was applied to a classified image layer consisting purely of safflower plants to increase safflower seedling detection accuracy (Fig. 2). The OBIA algorithm consisted of two main parts as described below:

#### Part I, safflower plant classification

The RGB orthomosaic image was segmented using the chessboard segmentation process to create single pixel objects which were classified as safflower plant objects if their green ratio was > 0.36. The green ratio is the proportion of the green waveband over the total of red, green and blue wavebands:

$$Green\;ratio = \frac{{\left[ {Green} \right]}}{{\left[ {Red} \right] + \left[ {Green} \right] + \left[ {Blue} \right]}}$$

Remaining objects were classified as background/soil. Plant objects were then merged and resulting objects with sizes < 30 pixels were excluded as background/soil.

#### Part II, template matching

**Fig. 3:**
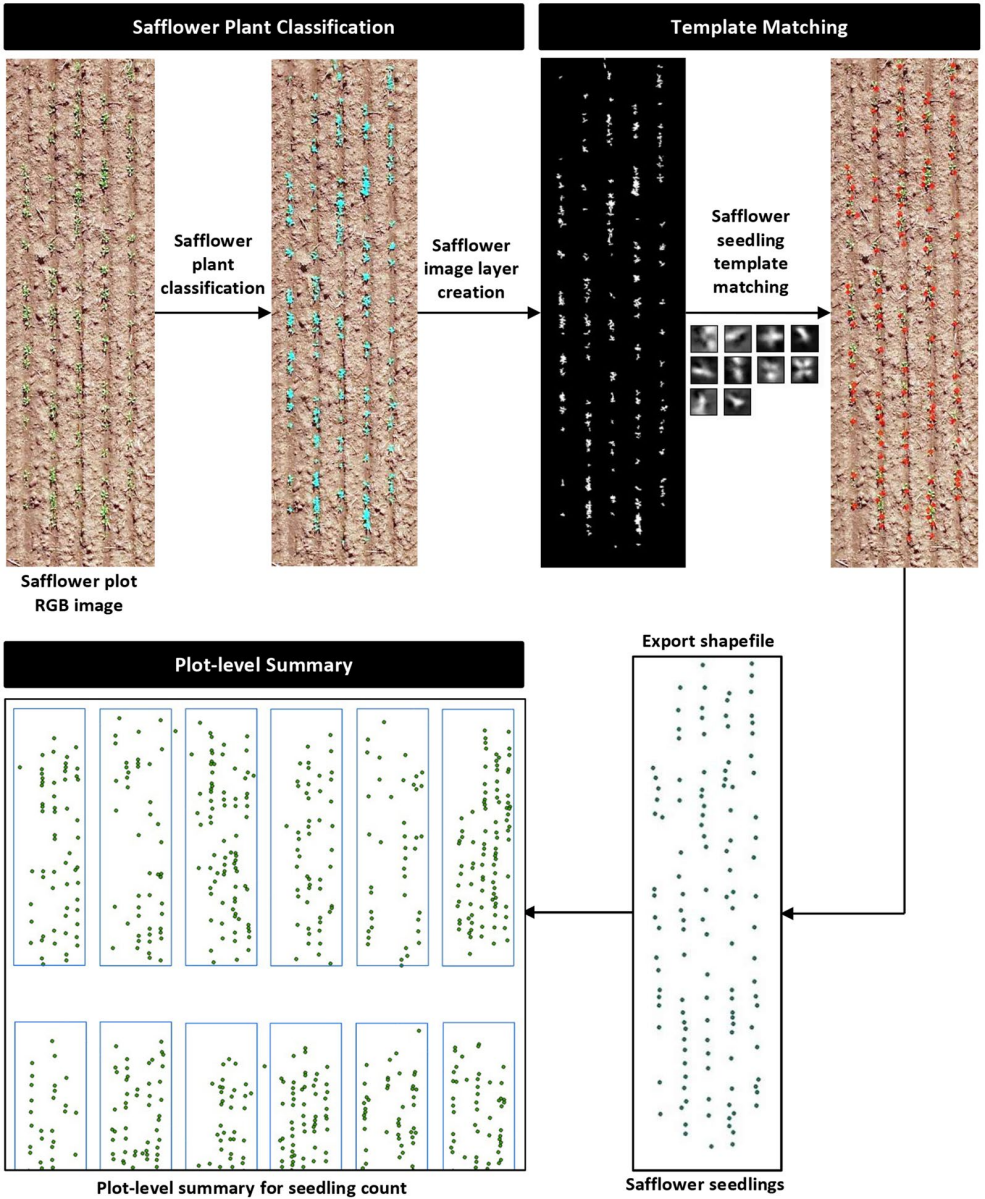
Graphical overview of the image analysis pipeline for safflower seedling count estimation. Examples of the primary outputs in the pipeline are presented at the plot level. Classified safflower plants (indicated in teal) are subjected to a grouped seedling template matching and detected seedlings (indicated in red) are exported as a point shapefile into ArcGIS Pro software for plot-level summary (plots outlined in blue) of seedling counts

The resulting safflower plant objects were segmented again using the chessboard segmentation process to create single pixel objects. The visible green channel layer containing the segmented plant objects was used to create an image layer named “safflower image layer”. The grouped template for safflower seedling was then applied to this safflower image layer and matches with a correlation coefficient of > 0.36 were classified as safflower seedling. The remaining matches were excluded as background i.e. soil, weeds or non-safflower plants. Further fine-tuning of the template matching accuracy was achieved by optimising the template matching stringency/threshold. Finally, safflower seedlings were exported as a point shapefile. Primary outputs from the OBIA algorithm are shown in Fig. 3.

### Data analysis

A field plot map was generated in ArcGIS Pro version 2.1 (https://www.arcgis.com/) and the polygons/rectangular borders were sized to the experimental plot dimension of 5 m × 1 m. The safflower seedlings point shapefile was imported into ArcGIS Pro and the plot map was used to summarise the total safflower seedlings count per plot and plant density (plants/m^2^) for each plot (Fig. 3). The accuracy of the OBIA algorithm was evaluated by comparing plant counts obtained by manual counting in the field (manual) to those estimated by the algorithm (digital) for 300 plots each from 2017 and 2018 field experiments. Accuracy metrics such as coefficient of determination (R^2^), mean absolute error (MAE) and root-squared mean error (RSME) were computed in Microsoft Excel. A higher accuracy is represented by a higher R^2^ score and lower values for both MAE and RSME.

## Results

### Template matching algorithm optimisation

Due to the diverse safflower genotypes and heterogenous germination present in the field experiments, a large number of sample patches (1000) representing safflower seedlings at growth stages of 2–4 leaves were used to generate the safflower seedling template. Initial testing showed that a template generated using images in the green channel had better quality (correlation coefficient, R = 0.294) compared to those generated using either the red (R = 0.283) or blue (R = 0.223) channels. However, as evidenced by the low R scores, template quality was low, most likely due to the large number of sample patches with contrasting morphological characteristics. As such, a two-step optimization strategy was employed to improve the template quality. The first step involved generating the safflower seedling template from a classified image layer in the green channel consisting purely of safflower plants (Fig. 3). This resulted in a 55% improvement in the template quality (R = 0.458), possibly due to an absence of background noise or foreign objects with similar properties to the target (e.g. weeds) in the sample patches. The second step involved generating grouped template types, as opposed to a single template for safflower seedlings. Results indicated that appreciable gains in template quality were observed when transitioning from single to grouped template types with 5 (R = 0.584), 10 (R = 0.628) and 15 (R = 0.631) subgroups. The grouped template with 10 subgroups was selected for further optimisation as improvement in template quality was negligible (< 0.05%) with 15 subgroups which entails a higher computational cost and longer processing time in template matching.

**Fig. 4:**
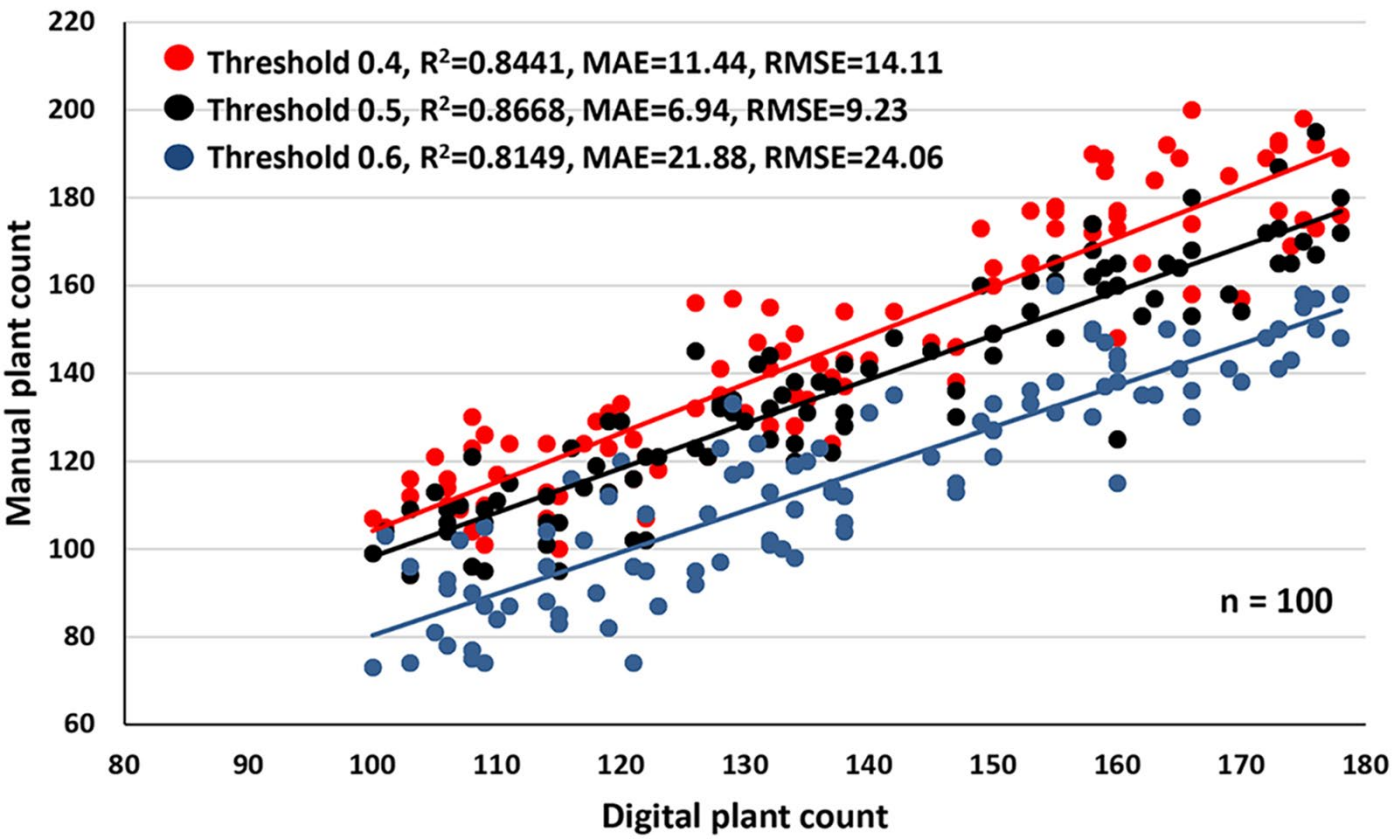
Performance of plant count estimation under different template matching stringencies. Template matching thresholds of 0.4, 0.5 and 0.6 were evaluated on 100 plots from the 2017 field experiment. Estimated (digital) plant counts were compared to manual plant count

The performance of the template matching algorithm was evaluated initially on 100 plots from the 2017 field experiment (Fig. 4). The effect of three template matching stringencies (thresholds = 0.4, 0.5 and 0.6; threshold of 1.0 being a perfect match) on performance was tested. Safflower seedling count estimated with a template matching threshold of 0.5 had the best accuracy (R^2^ = 0.8668, MAE = 6.94, RSME = 9.23) compared to a threshold of 0.4 (R^2^ = 0.8441, MAE = 11.44, RSME = 14.11) or a threshold of 0.6 (R^2^ = 0.8149, MAE = 21.88, RSME = 24.06) (Fig. 4). These results suggest that low template matching stringencies will likely result in an overestimation of plant count, whereas underestimation is expected under high stringencies. A template matching stringency of 0.5 was selected as the optimum value for further testing.

### Plant count estimation accuracy

**Fig. 5:**
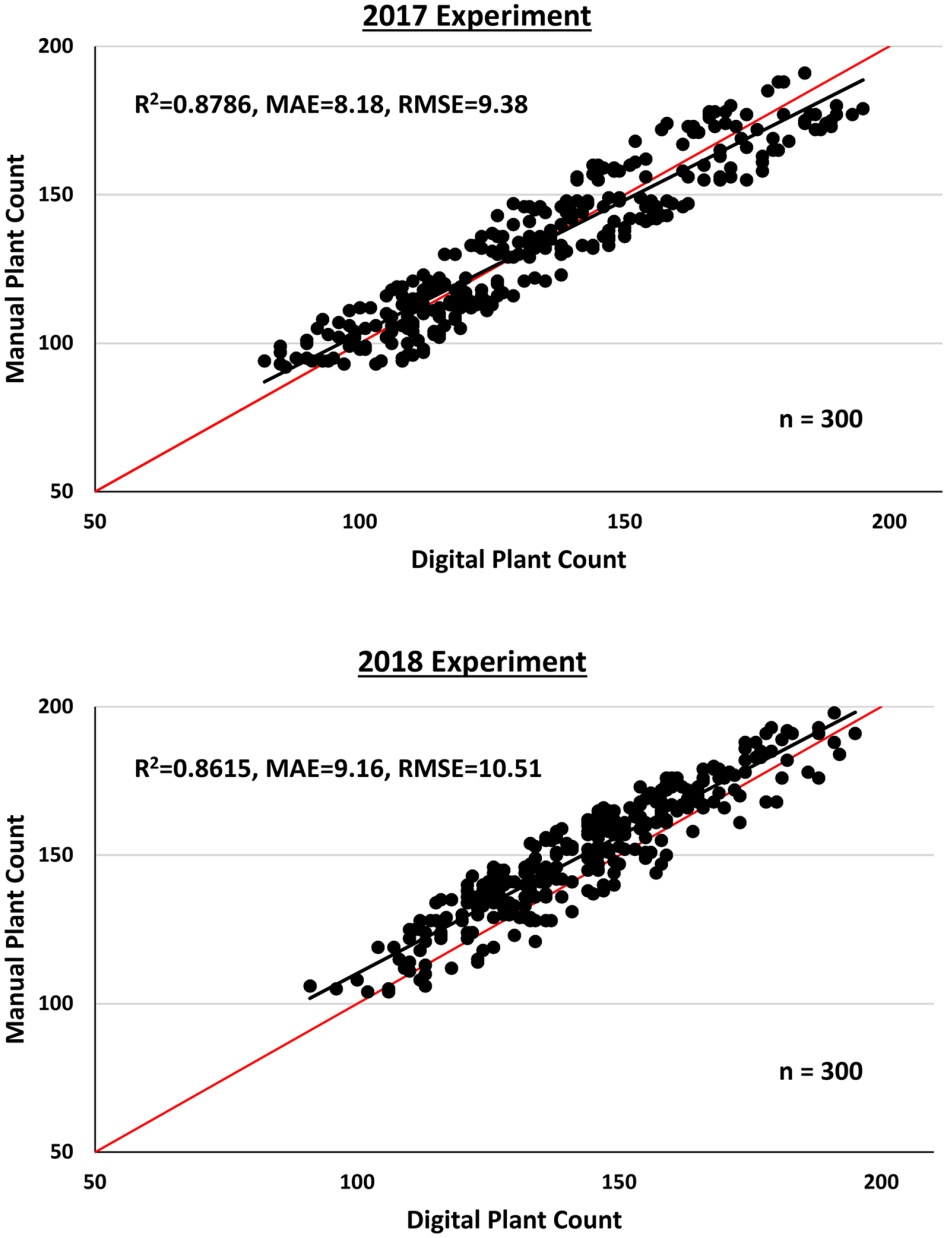
Performance of plant count estimation for field experiments. Plant count was estimated (digital) for 300 plots each for 2017 and 2018 experiments and compared to those obtained by manual counting. Red line indicates the 1:1 line

**Fig. 6:**
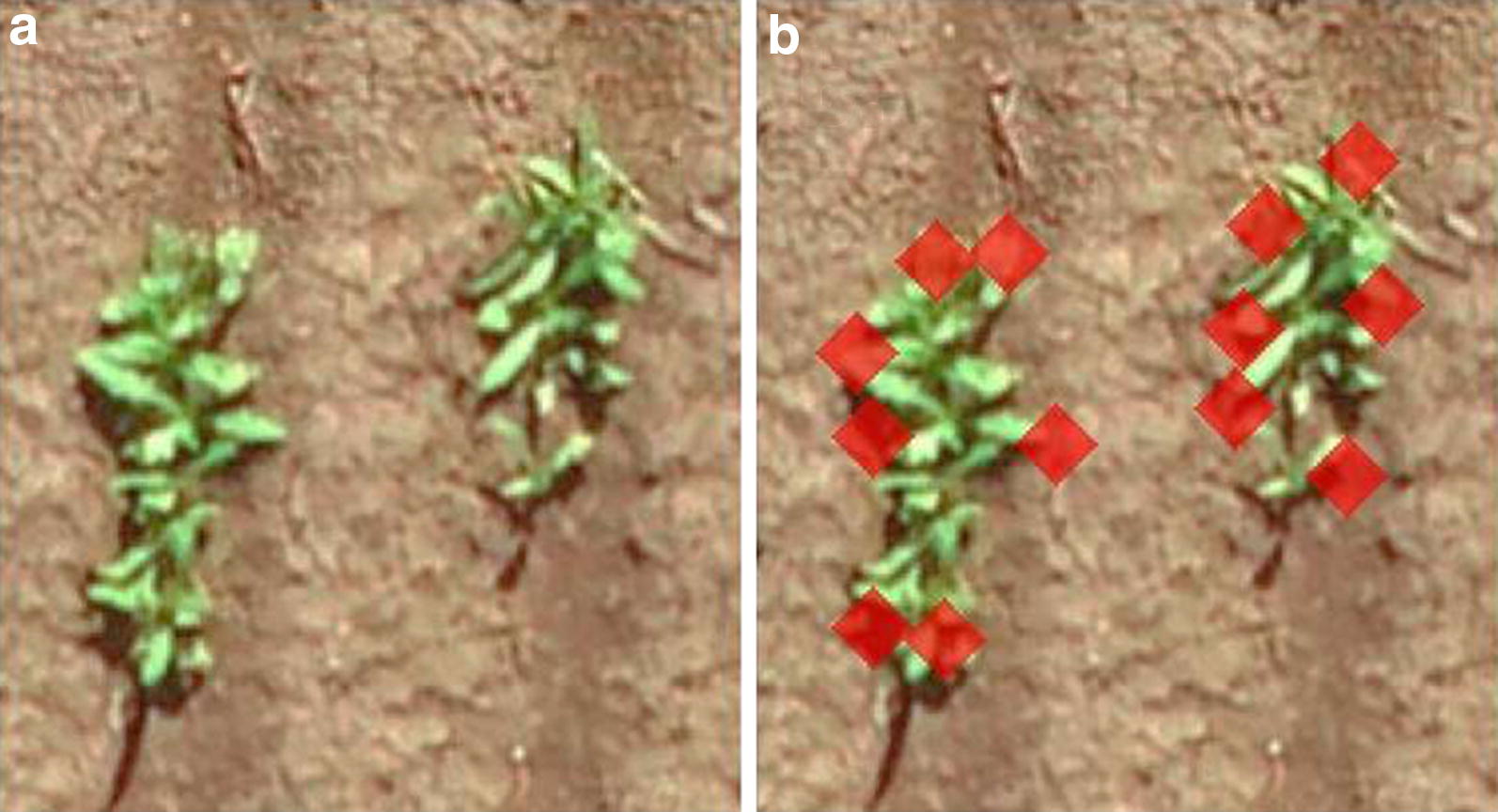
Safflower seedling detection over areas with high plant overlap. Magnified views (× 1.5) of an area with safflower seedling clusters before (**a**) and after (**b**) safflower seedling detection. Hits following template matching are indicated by red diamonds (13 plants). In contrast, manual counting based on image is 17 plants

The performance of the optimised OBIA algorithm for plant count estimation was tested and validated on an additional 300 plots each from the 2017 and 2018 field experiments (Fig. 5). For the 2017 field experiment, estimated safflower seedling counts correlated closely with counts obtained by manual counting (R^2^ = 0.8786, MAE = 8.18, RSME = 9.38). Similar results were obtained from the 2018 experiment (R^2^ = 0.8615, MAE = 9.16, RSME = 10.51), thus validating the high accuracy of the plant count estimation. In both experiments, a wide range of plant counts were observed, highlighting the diverse safflower genotypes and heterogenous germination present in each experiment. The results also suggest that the OBIA algorithm was effective even when contrasting growth stages of 2 to 4 leaves were present within and across safflower genotypes. Closer inspection showed that the performance of the algorithm degrades slightly (over- or under-estimation) for areas with tight clusters of safflower seedlings (Fig. 6), probably due to the complex situation caused by high overlaps between plants.

## Discussion

Recent studies have demonstrated the capability of UAV platforms for plant count estimation using RGB imagery in maize [14], cotton [15], potato [16], wheat [7] and rapeseed [8]. For larger plants with uniform distribution and wider spacing such as maize, cotton and potato, the image analysis process is relatively simpler as plants are typically represented as individual objects after image segmentation [14–16]. However, for overlapping crops such as wheat and rapeseed, spectral information alone is insufficient and additional features are required to estimate plant count using regression or machine learning models [7, 8]. This makes data analysis not only computationally challenging, but also technically difficult, often requiring expert guidance in feature extraction. For example, in rapeseed up to 15 morphological features were evaluated and only three features were selected for use in a multi-regression model for seedling count estimation [8]. Furthermore, features selected for a prediction model are likely specific to the crop species and growth stage [7, 8], thus extensive optimisation is required to apply the same model to other crop species.

In contrast, the OBIA algorithm based on template matching for safflower seedlings presented in this study is relatively simple to implement due to a more visual approach requiring the operator to select sample patches from RGB imagery representing the targets of interest. In addition, results generated by template matching are easy to interpret, as positive hits can be overlayed on top of existing RGB images, thus offering a quick visual verification of outputs. Perhaps the most attractive feature of the OBIA algorithm is its potential to be deployed onto crops with heterogenous germination and contrasting growth stages, something often seen in field experiments with diverse genotypes or different treatments such as varying watering or nutrient regimes. In both wheat and rapeseed studies, the growth stage had a significant impact on the performance of the seedling count models [8, 17]. In comparison, results in this study indicate that the OBIA algorithm was able to detect safflower seedlings at various growth stages from 2 to 4 leaves, largely due to the grouped template (10 subgroups) approach which accounts for different seedling growth types. Combined with the strategy of using classified image layers containing only safflower plant objects as input for template matching, seedling count estimation correlated closely with manual counts for both 2017 (R^2^ = 0.8786) and 2018 (R^2^ = 0.8615) experiments. These results are comparable to the performance of seedling count models published for other crop species [7, 8, 14–17].

A common challenge in seedling count models is the separation of plant from background, especially when green weeds are present. Methods employing classification based on colour [18] and shape [19] were successful in weed identification and separation from plants. The ability to generate an image layer consisting purely of safflower plant objects was crucial to the success of the OBIA algorithm in this study. Although weeds were well-controlled in our field experiments, the first part of the OBIA algorithm (plant classification) can be extended to incorporate a myriad of segmentation and classification algorithms to achieve satisfactory plant separation from the background in the event of a weed infestation using spectral and spatial information, for example combining data from multiple sensors such as RGB, multispectral and LiDAR.

Although germination rates varied significantly between safflower genotypes and across experiments, the OBIA algorithm performed well across a wide range of seedling densities. Results from our study indicate that the performance of seedling count estimation reduces slightly over areas with high plant overlaps, such as in tight clusters. As such, further studies are required to determine the performance of seedling count estimation under high plant densities, particularly for different safflower cultivation practises or when adopting this method for other densely sown crop species. For crops with small seedling and high overlaps, assuming a fairly uniform germination and growth stage, machine learning approaches using spectral, texture and morphological features may be better suited for seedling count estimation [7, 8, 17]. However, even for seedling count models based on machine learning approaches, complex situations arising from high plant overlaps remain a challenge [17]. Nevertheless, the OBIA algorithm developed for safflower seedling count estimation will facilitate high-throughput and reliable data collection for field experiments using UAV-acquired RGB imagery. Furthermore, this method could find wider application in other crop species, particularly dicot plants with seedlings similar to safflower. This will result in significant time and cost savings for large-scale agronomic and breeding field experiments with diverse genotypes or treatments where seedling count is a required phenotypic observation.

## Conclusions

A method for safflower seedling count estimation at early stages based on UAV-acquired RGB imagery was developed and validated in this study. The method employs an OBIA algorithm based on template matching for safflower seedling detection. Results indicate that the OBIA algorithm performed well even when seedlings with contrasting growth stages were present and has the potential to be adopted for use in other crop species. This method will facilitate high-throughput data collection for field experiments using UAV platforms.

## Data Availability

All data generated or analysed during this study are included in this published article.

## References

[1] Yang G, Liu J, Zhao C, Li Z, Huang Y, Yu H, Xu B, Yang X, Zhu D, Zhang X (2017). Unmanned aerial vehicle remote sensing for field-based crop phenotyping: current status and perspectives. Front Plant Sci.

[2] Zhang C, Kovacs JM (2012). The application of small unmanned aerial systems for precision agriculture: a review. Precis Agric.

[3] Holman F, Riche A, Michalski A, Castle M, Wooster M, Hawkesford M (2016). High throughput field phenotyping of wheat plant height and growth rate in field plot trials using UAV based remote sensing. Remote Sens.

[4] Ludovisi R, Tauro F, Salvati R, Khoury S, Mugnozza Scarascia G, Harfouche A (2017). UAV-based thermal imaging for high-throughput field phenotyping of black poplar response to drought. Front Plant Sci.

[5] Madec S, Baret F, de Solan B, Thomas S, Dutartre D, Jezequel S, Hemmerlé M, Colombeau G, Comar A (2017). High-throughput phenotyping of plant height: comparing unmanned aerial vehicles and ground LiDAR estimates. Front Plant Sci.

[6] Watanabe K, Guo W, Arai K, Takanashi H, Kajiya-Kanegae H, Kobayashi M, Yano K, Tokunaga T, Fujiwara T, Tsutsumi N (2017). High-throughput phenotyping of sorghum plant height using an unmanned aerial vehicle and its application to genomic prediction modeling. Front Plant Sci.

[7] Jin X, Liu S, Baret F, Hemerlé M, Comar A (2017). Estimates of plant density of wheat crops at emergence from very low altitude UAV imagery. Remote Sens Environ.

[8] Zhao B, Zhang J, Yang C, Zhou G, Ding Y, Shi Y, Zhang D, Xie J, Liao Q (2018). Rapeseed seedling stand counting and seeding performance evaluation at two early growth stages based on unmanned aerial vehicle imagery. Front Plant Sci.

[9] Food and agriculture data, FAOSTAT. Food and Agriculture Organisation of the United Nations. 2016. http://www.fao.org/faostat/en/#data/QC. Accessed 13 Dec 2018.

[10] Wood CC, Okada S, Taylor MC, Menon A, Matthew A, Cullerne D, Stephen SJ, Allen RS, Zhou XR (2018). Seed-specific RNAi in safflower generates a superhigh oleic oil with extended oxidative stability. Plant Biotechnol J.

[11] SHO safflower case study. In: biobased oils. Commonwealth Scientific and Industrial Research Organisation. 2018. https://www.csiro.au/en/Research/AF/Areas/Plant-Science/Bio-based-oils/SHO-safflower. Accessed 13 Dec 2018.

[12] Collis B. Hopes for new industry from safflower ‘reinvention’. In: GroundCover. Grains Research and Development Corporation. 2018. https://grdc.com.au/resources-and-publications/groundcover/groundcover-133-march-april-2018/hopes-for-new-industry-from-safflower-reinvention. Accessed 13 Dec 2018.

[13] Safflower northern region. In: GrowNotes. Grains Research and Development Corporation. 2017. https://grdc.com.au/resources-and-publications/grownotes/crop-agronomy/safflowergrownotesnorth. Accessed 13 Dec 2018.

[14] Gnädinger F, Schmidhalter U (2017). Digital counts of maize plants by unmanned aerial vehicles (UAVs). Remote Sens.

[15] Chen R, Chu T, Landivar JA, Yang C, Maeda MM (2018). Monitoring cotton (*Gossypium hirsutum* L.) germination using ultrahigh-resolution UAS images. Precis Agric.

[16] Sankaran S, Quirós JJ, Knowles NR, Knowles LO (2017). High-resolution aerial imaging based estimation of crop emergence in potatoes. Am J Potato Res.

[17] Liu S, Baret F, Andrieu B, Burger P, Hemmerlé M (2017). Estimation of wheat plant density at early stages using high resolution imagery. Front Plant Sci.

[18] Gée C, Bossu J, Jones G, Truchetet F (2008). Crop/weed discrimination in perspective agronomic images. Comput Electron Agric.

[19] Swain KC, Nørremark M, Jørgensen RN, Midtiby HS, Green O (2011). Weed identification using an automated active shape matching (AASM) technique. Biosyst Eng.

